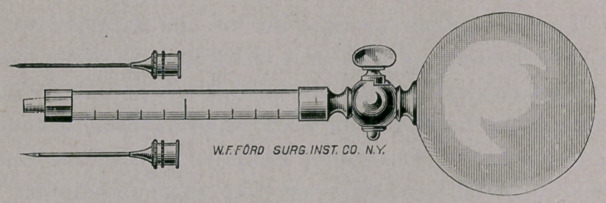# Koch’s Syringe

**Published:** 1891-02

**Authors:** 


KOCH'S SYRINGE.

Koch's Syringe for Hypodermic Injections, an illustration of which we present herewith, is likely to be in demand, not only for the use of his anti-tubercular treatment, but in other conditions for which hypodermic medication is indicated. It has the advantage over the piston syringe of being capable of absolute cleanliness. The solutions used come into contact only with the glass measuring tube and the metallic needle, and these can readily be rendered aseptic with heat and absolute alcohol. Instead of a piston, a rubber ball furnishes the means for expelling the solution, and a stopcock prevents any pressure upon the contents until everything is adjusted and ready for the injection.

Stoddart Brothers, Surgical Instrument Specialists, of 84 Seneca street, Buffalo, N. Y., are prepared to furnish the syringes at the net price of $2.50. Attention is called to their advertisement on page XXIV. of the advertising columns of this Journal.

## Figures and Tables

**Figure f1:**